# The Assessment of Reconstructive Urology-Associated Google Search Trends During COVID-19

**DOI:** 10.7759/cureus.18305

**Published:** 2021-09-26

**Authors:** Cem Kezer

**Affiliations:** 1 Urology, Gunesli Erdem Hospital, Istanbul, TUR

**Keywords:** reconstructive urology, world pandemic, public interest, google trends healthcare, covid-19

## Abstract

Introduction: To demonstrate public interest in reconstructive urology during the COVID-19 pandemic by using Google Trends (GT).

Methods: The study was conducted between August 1 and August 11, 2021. A total of 18 terms related to reconstructive urology were determined. Public interest in all terms were evaluated with the GT application using the filters ‘worldwide’, ‘all categories’, and ‘web search’. To determine public interest in reconstructive urology during the COVID-19 pandemic, three 12-week periods following the declaration of COVID-19 (March 11 to June 4, 2020, June 5 to August 29, 2020, and August 30 to November 23, 2020) were compared with the same periods in the past four years (2016-2019).

Results: Comparisons of March 11 to June 4, 2020, and the same days in the previous four years revealed that total public attention to reconstructive urology significantly declined (−16.2%, p=0.035). In the comparison of the second 12-week duration (June 5 to August 29, 2020, vsJune 5 to August 29, 2016-2019), only the bladder augmentation term had significantly lower search frequency during COVID-19 (−46.3%, p=0.043), but buried penis (50.3%, p=0.001), urinary incontinence (15.3%, p=0.001), and stress urinary incontinence (21.7%, p=0.001) keywords had significantly higher inquiries. The comparison of the third 12-week period searches for only urinary incontinence significantly increased (p=0.001).

Conclusion: Present study showed that public interest in reconstructive urology significantly reduced in the first 12 weeks after COVID-19 was declared a pandemic. However, public attention to reconstructive urology reached similar levels after 12 weeks from the beginning of COVID-19. Additionally, the term urinary incontinence was searched statistically more frequently during the COVID-19.

## Introduction

The novel coronavirus infection (COVID-19) was first detected at the end of 2019 in South Asia, and COVID-19 spread to all continents in a short time. Although COVID-19 could present like a common cold or flu-like syndrome, the relationship between COVID-19 and pneumonia and the severe acute respiratory syndrome was proven [1]. According to the World Health Organization (WHO), COVID-19 affected almost 250 million people, and 4.5 million individuals died due to COVID-19 and COVID-19-associated complications [2]. In early March 2020, WHO accepted COVID-19 as a pandemic, and numerous governments declared social isolation, transportation restrictions, and quarantine to prevent quick transmission of COVID-19. Additionally, the number of outpatient clinics were reduced and surgical procedures were deferred. Difficulties in accessing the health system during COVID-19 led people to obtain knowledge about their complaints and disorders from television, books, and the internet.

Search engines are chosen by many people to reach knowledge on the internet. Although there are many search engines in the market, Google Search (Google, Inc., Mountain View, California, USA) is the most preferred and is the preference of 90% of all internet users. Google Trends (GT) was developed to present data about how often a phrase or word is searched for, in which languages, and in which country by internet users in Google searches [3]. During the Zika virus epidemic, Teng et al. used GT to analyze public interest about the epidemic and concluded that GT provides information about the progress of a pandemic [4]. 

Although public interest in different health problems during the COVID-19 pandemic was evaluated in previous research by using GT, there is no study that evaluates public attention to reconstructive urology. In the present study, we aimed to analyze public attention to reconstructive urology during the COVID-19 pandemic.

## Materials and methods

The study was conducted between August 1 and August 11, 2021. A total of 18 terms (urethroplasty, urethral stricture, urethrotomy, hypospadias, buried penis, uterine prolapse, bladder prolapse, cystocele, rectocele, bladder augmentation, vesicourethral reflux, ureteroneocystostomy, pyeloplasty, vesicovaginal fistula, urinary incontinence, male sling, stress urinary incontinence, and artificial urinary sphincter) related to reconstructive urology were determined. Public interest in all terms were evaluated with the GT application using the filters ‘worldwide’, ‘all categories’, and ‘web search’.

Google Trends

Google Trends is an application to obtain knowledge about any term among analogous keywords from all inquiries done by using Google search in a particular time period. In addition, GT provides information about the most attractive keywords and issues to make decisions before creating a website. The results of internet research can be obtained and noted from the GT official website (https:// trends.google.com). For any keyword, the score on GT ranges between 0 and 100 (lowest to highest) and a higher GT score for the inquired keyword demonstrates higher relative attention.

To determine public interest in reconstructive urology during the COVID-19 pandemic, three 12-week periods following the declaration of COVID-19 (March 11 to June 4, 2020, June 5 to August 29, 2020, and August 30 to November 23, 2020) were compared with the same periods in the past four years (2016-2019). The World Health Organization declared COVID-19 a pandemic on March 11; thus, the present study accepted it as the starting date of the study. In this study, no patient data were analyzed; thus, Institutional Ethics Committee approval was not needed.

Statistical analysis

In this study, IBM SPSS v.20 (IBM Corp., Armonk, NY) was used for statistical analyses. To analyze continuous parameters, arithmetic means ± standard deviations were used. The Kolmogorov Smirnov test was used to determine the normality assumption. To present differences between groups, the Wilcoxon test and paired samples T-test were applied. Statistical significance was accepted as p<0.05.

## Results

Comparisons of March 11 to June 4, 2020, and the same days in the previous four years revealed that total public attention to reconstructive urology significantly declined (-16.2%, p=0.035). Inquiries about 11 of 18 keywords including urethroplasty, urethrotomy, hypospadias, cystocele, rectocele, bladder augmentation, vesicourethral reflux, pyeloplasty, vesicovaginal fistula, male sling, and artificial urinary sphincter were significantly lower in comparison to the same period between 2016 and 2019. In contrast, only searches for the urinary incontinence term were significantly increased. Additionally, searches of keywords including urethral stricture, buried penis, uterine prolapse, bladder prolapse, ureteroneocystostomy, and stress urinary incontinence were similar during the COVID-19 era and the previous four years.

In the comparison of the second 12-week duration (June 5 to August 29, 2020 vs June 5 to August 29, 2016-2019), public interest in reconstructive urology terms was similar (p=0.645). Only the bladder augmentation term had significantly lower search frequency during COVID-19 (-46.3%, p=0.043), but buried penis (50.3%, p=0.001), urinary incontinence (15.3%, p=0.001) and stress urinary incontinence (21.7%, p=0.001) keywords had significantly higher inquiries. Public interest in the remaining 14 terms was comparable between June 5 and August 29, 2020 and June 5 to August 29, 2016-2019.

The comparison of the third 12-week period (August 30 to November 23, 2020, and the same period between 2016 and 2019) showed that public attention was similar (2.7%, p=0.765). In addition, 17 of 18 keywords had comparable search frequency during COVID-19 and the previous four years. Only, searches for urinary incontinence significantly increased between August 30 and November 23 during COVID-19 (9.4%, p=0.001) (Table 1).

**Table 1:** Analysis of Google Trends search terms by period

	March 11 – June 4				June 5 – August 29				August 30 – November 23			
	2020	2016-2019	%change	P-value	2020	2016-2019	%change	P-value	2020	2016-2019	%change	P-value
Urethroplasty	7.1±11.7	15.5±19.1	−54.2	0.001	15.6±11.9	16.8±12.4	−7.1	0.346	12.6±13.8	13.3±17.4	−5.3	0.594
Urethral stricture	23.4±16.9	22.2±18.0	5.4	0.645	28.6±18.6	23.5±19.4	21.7	0.059	31.0±19.6	28.3±20.9	9.5	0.371
Urethrotomy	8.2±16.0	14.7±22.7	−44.2	0.010	11.8±6.7	12.5±8.9	−5.6	0.672	12.1±17.9	11.2±17.3	8.0	0.795
Hypospadias	18.8±7.4	24.4±11.8	−22.9	0.001	20.6±11.5	18.9±9.7	9.0	0.126	40.2±25.4	39.4±18.5	2.1	0.687
Buried penis	17.8±18.8	17.5±20.6	1.7	0.760	21.2±16.4	14.1±9.8	50.3	0.001	20.1±16.4	19.5±12.6	3.1	0.476
Uterine prolapse	24.1±14.1	27.2±16.2	−11.4	0.059	36.1±12.3	34.3±13.4	5.2	0.568	36.5±18.3	35.5±20.5	2.8	0.556
Bladder prolapse	20.6±11.9	24.4±15.0	−15.6	0.055	30.8±15.6	28.9±14.3	6.6	0.469	24.8±14.3	24.7±16.2	0.4	0.872
Cystocele	24.7±14.5	34.0±18.4	−27.3	0.001	35.8±12.3	36.1±11.4	−0.1	0.876	35.2±16.7	35.8±18.5	−1.7	0.796
Rectocele	23.2±15.6	35.2±19.5	−34.1	0.001	35.7±9.7	34.1±8.6	4.7	0.366	36.4±18.5	37.4±20.2	−2.7	0.645
Bladder augmentation	2.8±11.4	6.2±12.4	−54.8	0.001	2.9±9.1	5.4±8.7	−46.3	0.043	3.5±10.9	4.7±14.7	−25.5	0.807
Vesicourethral reflux	15.9±18.0	20.2±20.6	−21.3	0.032	17.2±8.7	17.8±9.1	−3.4	0.789	18.3±16.2	17.2±18.9	6.4	0.730
Ureteroneocystostomy	4.2±9.6	4.6±8.7	−8.7	0.368	2.1±6.5	2.6±7.2	−19.2	0.387	4.1±10.2	3.6±9.4	13.9	0.465
Pyeloplasty	13.5±15.0	18.5±18.8	−27.0	0.013	15.2±11.4	16.3±12.4	−6.7	0.481	23.9±22.0	21.5±20.4	11.2	0.757
Vesicovaginal fistula	9.6±12.4	13.2±11.5	−27.3	0.001	9.1±9.6	8.8±10.2	3.4	0.697	11.5±18.6	11.6±21.2	−0.8	0.970
Urinary incontinence	65.6±8.5	52.2±24.1	25.7	0.001	73.2±11.2	63.5±12.3	15.3	0.001	67.4±8.5	61.6±12.0	9.4	0.001
Male sling	5.4±7.2	10.2±9.8	−47.0	0.001	9.1±8.7	9.7±7.6	−6.2	0.453	7.8±9.3	8.3±10.7	−6.0	0.378
Stress urinary incontinence	16.4±9.7	16.2±12.7	1.2	0.684	20.2±11.5	16.6±8.9	21.7	0.001	15.4±9.7	17.2±8.6	−10.5	0.256
Artificial urinary sphincter	6.4±9.6	10.2±7.6	−37.2	0.021	6.2±8.7	5.9±7.9	5.1	0.867	7.2±8.4	6.4±8.1	12.5	0.654
Total	17,1±11.2	20.4±13.4	−16.2	0.035	21.7±8.5	21.0±9.1	3.3	0.645	22.7±9.8	22.1±9.2	2.7	0.765

## Discussion

Resources that patients refer to for health information have dramatically changed in the last two decades, and internet sources became an important way to get information [5]. Previous reports stated that one in every two people use Google to obtain information from the internet, and almost 25% of these people research in English [6]. During the COVID-19 era, more people started to use the internet instead of attending hospitals or doctor visits. In the present study, we aimed to demonstrate public interest in reconstructive urology by using GT. Public interest in reconstructive urology decreased in the first 12-weeks at the beginning of the pandemic, but after 12 weeks public attention about reconstructive urology reached pre-COVID levels. In addition, public interest in the urinary incontinence term was significantly higher over the three 12-week periods.

Differences in public interest between the COVID-19 era and the pre-COVID-19 era were investigated in previous studies. Guzman and Barbieri found a significant reduction in the public interest in cosmetic and dermatologic diseases in the first 15 days of the COVID-19 pandemic. Also, Guzman and Barbieri added that public attention to cosmetic and dermatologic disease reached pre-COVID levels after one month [7]. In a different study, Kardes et al. investigated the public interest in rheumatological illness during the COVID-19 pandemic and found a significant decrease in social interest in rheumatological diseases in the first 12 weeks of the COVID-19 pandemic [8]. In accordance with the aforementioned studies, we found significantly lower public interest in reconstructive urology in the first 12 weeks at the beginning of the COVID-19 pandemic. However, public attention to reconstructive urology reached pre-COVID levels after 12 weeks.

Every term has a distinctive search number according to Google search. In our study, we found that public interest in urinary incontinence was significantly higher in the three 12-week periods after the declaration of the COVID-19 pandemic. We have two explanations for this result. First, the term urinary incontinence is more likely to be known by the public than other specific terms such as pyeloplasty, urethrotomy, artificial urinary sphincter, etc. Second, the incidence of urinary incontinence is significantly higher than other diseases including urethral stricture, ureteropelvic junction obstruction, or vesicovaginal fistula. Some authors stated that almost 25% of the population experience urinary incontinence during their lifespan [9].

Late diagnosis and also the late treatment of reconstructive urologic disorders due to COVID-19 could be associated with progression of the disease, increments in morbidity and mortality, and increases in healthcare budgets. Waldstein and Katzel demonstrated that undiagnosed and untreated hypertension was a risk factor for worse cognitive brain activity, and the authors stated the unfavorable effect of delayed hypertension diagnosis on cognitive brain functions [10]. Also, McKinley et al. found negative correlations between delays in cardiac disease diagnosis and cardiac treatment benefit [11]. However, there is no study that evaluated the impact of COVID-19 on the diagnosis and management of reconstructive urologic disease, which may be analyzed in further studies.

Interestingly, the buried penis term was searched for at significantly higher rates in the second 12-week period during COVID-19. Yuksel and Cakmak investigated the sexual attitudes of women and their partners and found significant increments in sexual intercourse with significantly lower sexual satisfaction [12]. In another study, Zattoni et al. found an increased interest in pornography during the COVID-19 pandemic [13]. We believe that increased interest in sexual activity during extended periods of time spent at home and growing interest in pornography during the COVID-19 pandemic are associated with higher public attention about the buried penis term.

Even though the present study is the first to evaluate public interest in reconstructive urology by using GT, our study has some limitations. First, there are different search engines other than Google. However, almost 90% of internet users choose the Google search engine during their internet research. Second, we only included keywords in the English language. We think that analyzing more than one language would be complex and confusing. In addition, the English language is the most used language in Google search. Also, we investigated only the name and treatment modalities in reconstructive urology, not patient complaints which could be a subject for further studies.

## Conclusions

In conclusion, the present study showed that public interest about reconstructive urology significantly reduced in the first 12 weeks after COVID-19 was declared a pandemic. However, public attention about reconstructive urology reached similar levels after 12 weeks from the beginning of COVID-19. Additionally, the term urinary incontinence was searched for statistically more frequently during the COVID-19. Therefore, surgeons interested in reconstructive urology should return to the practice of reconstructive urology as well as fighting COVID-19.
